# Cross-Cultural Adaptation and Psychometric Properties of the French Version of the EXIT to Measure Women’s Experiences of Induction of Labor

**DOI:** 10.3390/jcm11144217

**Published:** 2022-07-20

**Authors:** Candy Guiguet-Auclair, Marion Rouzaire, Anne Debost-Legrand, Sigrid Dissard, Manon Rouille, Amélie Delabaere, Denis Gallot

**Affiliations:** 1 Université Clermont Auvergne, CHU Clermont-Ferrand, CNRS, Institut Pascal, F-63000 Clermont-Ferrand, France; alegrand@chu-clermontferrand.fr (A.D.-L.); adelabaere@chu-clermontferrand.fr (A.D.); 2 Department of Public Health, CHU Clermont-Ferrand, F-63000 Clermont-Ferrand, France; 3 Department of Obstetrics and Gynecology, CHU Clermont-Ferrand, F-63000 Clermont-Ferrand, France; mrouzaire@chu-clermontferrand.fr (M.R.); sigrid.dissard@etu.uca.fr (S.D.); mrouille@chu-clermontferrand.fr (M.R.); dgallot@chu-clermontferrand.fr (D.G.); 4 Réseau de Santé Périnatale d’Auvergne, CHU Clermont-Ferrand, F-63000 Clermont-Ferrand, France; 5 Translational Approach to Epithelial Injury and Repair, Faculty of Medicine, Université Clermont-Auvergne, CNRS 6293, INSERM 1103, GReD, F-63000 Clermont-Ferrand, France

**Keywords:** EXIT, induction of labor, patient-reported outcome measure, cross-cultural adaptation, psychometric properties

## Abstract

Background: In France, more than 20% of women require induction of labor (IOL), which can be psychologically and emotionally challenging for patients. It is important to assess how they feel about their IOL experiences. Our aim was to cross-culturally adapt and evaluate the psychometric properties of a French version of the EXIT to assess women’s experiences of IOL. Methods: The EXIT was cross-culturally adapted by conducting forward and backward translations following international guidelines. A cross-sectional study was conducted to assess the psychometric properties of the ten French EXIT items: data completeness, factor analysis, internal consistency, score distribution, floor and ceiling effects, inter-subscale correlations, convergent validity, and test–retest reliability. Results: The EXIT was successfully cross-culturally adapted to the French context and any IOL method. The results obtained from 163 patients requiring IOL showed good acceptability. Exploratory factor analysis resulted in a three-factor solution with subscales reflecting the experiential aspects of time taken to give birth, discomfort with IOL, and subsequent contractions. Good internal consistency (Cronbach’s alpha or Spearman correlation coefficients ranging from 0.55 to 0.84) and good test–retest reliability (intraclass correlation coefficients ranging from 0.66 to 0.85) for the three identified subscales were found. Conclusions: The ten-item French EXIT is a valid and reliable instrument for the self-assessment of women’s experiences of IOL in the three weeks following delivery for any method of IOL used. As a patient-reported outcome measure, it would allow the comparison of experiential outcomes across IOL studies in order to include women’s preferences in decisions regarding their care.

## 1. Introduction

In France, more than 20% of women are induced to give birth [1,2]. Induction of labor (IOL) is defined as a medical intervention designed to artificially induce uterine contractions, which leads to the progressive retraction and dilation of the uterine cervix, resulting in birth. Induction is intended for women who have not gone into labor, regardless of the state of the membranes. It should only be performed if it appears that in terms of health, the mother or the child will have a more favorable outcome than if the delivery had occurred later [3,4]. The medical indications for IOL are prolonged pregnancy [4,5,6], prelabor rupture of amniotic membranes [4,7], diabetes, suspected fetal macrosomia [8], multiple pregnancies [4], fetal growth restriction [4], hypertension and pre-eclampsia [4], and fetal pathologies requiring specific neonatal management [4]. The mode of induction is chosen according to the indication, the state of the membranes, the Bishop score, and the equipment available [9,10,11,12].

The IOL experience can be difficult for patients from a psychological and emotional perspective [13,14]. Depending on the parity and the state of the ripening of the cervix, induction may lead to a delay in labor that is more or less long and difficult for obstetricians to predict. In the case of an unfavorable cervix, the ripening methods used may cause pain without causing cervical dilatation. The device used may itself cause discomfort to the patient.

For these reasons, women’s experiences and satisfaction are increasingly evaluated in clinical studies comparing the effectiveness of induction methods [15,16,17,18,19,20,21]. Studying women’s preferences and feelings about their experiences of IOL could help to better consider them in decisions about their care.

Despite the frequency of this obstetrical situation, there are few studies on women’s perceptions of their IOL experiences, and the results of the studies are inconsistent. Furthermore, among these trials, no universal tool has been used to measure women’s experiences.

The EXIT (EXperiences of Induction Tool) was recently developed by three psychologist–researchers in a randomized controlled trial comparing early amniotomy with repeat vaginal prostaglandin administration [22]. It is a valid and reliable self-reporting instrument designed to measure the meaningful aspects of women’s experiences of IOL. Evidence was demonstrated for its construct validity in a three-component solution, internal consistency, internal convergent validity, and discriminant validity.

To our knowledge, no validated and reliable tool exists in France to assess women’s experiences of IOL. Thus, we performed a study to cross-culturally adapt and evaluate the psychometric properties of a French version of the EXIT. Initially developed to assess women’s experiences using prostaglandin vaginal gel for IOL, we also adopted this instrument for all methods of IOL.

## 2. Materials and Methods

This study was conducted in two phases: first, the original EXIT was cross-culturally adapted from English to French; then, the psychometric properties of the French version of EXIT were assessed. The test–retest reliability was notably explored as recommended in the original study [22].

### 2.1. The EXIT

The EXIT self-administrated questionnaire contains 10 items developed to capture the meaningful aspects of women’s experiences of IOL [22], which are rated on a 5-point Likert scale from 1 (strongly disagree) to 5 (strongly agree). Eight of these ten items comprise three subscales: ‘Time taken to give birth’ (2 items), ‘Discomfort with IOL’ (4 items), and ‘Experience of subsequent contractions’ (2 items). Scores for the subscales are obtained by calculating the mean of the individual scores of the items listed in the subscale. Five of the ten items have reverse scores (items 3, 4, 6, 7, and 10). One item is addressed only to women who underwent artificial rupture of membranes (ARM group). Three optional added single-item measures ask women about global satisfaction with the birth experience, the likelihood of choosing the same method of IOL again, and the likelihood of recommending the method of IOL to a friend or relative. Responses are rated on a 5-point Likert scale from 1 (strongly disagree) to 5 (strongly agree) for global satisfaction and from 1 (definitely not) to 5 (definitely) for the two other items. In addition, four optional items evaluate the IOL process, two quantitative items are rated on a 5-point Likert scale from 1 (definitely not) to 5 (definitely) (perceived adequate preparation for induction and perceived necessity of all medical procedures during birth), and two qualitative items explore women’s experiences of IOL and birth and their suggestions on ways to improve the experiences of other women undergoing IOL.

### 2.2. Translation and Cross-Cultural Adaptation of the French Version of EXIT

Items were translated from English into French and cross-culturally adapted to be relevant to the French context, following international guidelines for the adaptation of self-administered instruments [23,24]. The original EXIT was applied to only one method of IOL (prostaglandin vaginal gel). To adapt this tool to any IOL method, as recommended by Beckmann et al. [22], “after I first had the vaginal gel” was replaced with “after being induced”. Forward translations were independently made by three bilingual translators fluent in English, with French as their mother tongue, two of them being naïve to the outcomes measured. A multidisciplinary expert committee (composed of an obstetrician, a public health physician, and a methodologist) reviewed the three translations and edited a first consensus French version. Linguistic equivalence to the original English version was discussed. Then, two native English translators fluent in French and blinded to the original English version made a backward translation. The expert committee compared the source and target versions and resolved discrepancies. Item translation, semantic, idiomatic, cultural, experiential, and conceptual equivalents were discussed.

The consensus for the French version was pre-tested on a sample of twenty-one patients who required IOL in order to evaluate the comprehensibility of instructions and items. No difficulties in understanding or problems with completion were noticed. Consequently, the expert committee adopted this version as the pre-final cross-cultural adaptation (File S1). We named this version the EXIT-French.

The evaluation of the psychometric properties (given in detail below) was then conducted.

### 2.3. Study Design and Participants

A cross-sectional study was conducted from June 2020 to December 2021 at the University Hospital of Clermont-Ferrand. The study was approved by the French Committee for the Protection of Individuals Southeast VI (CPP Sud-Est VI Clermont-Ferrand, no. 2020/CE67, 14 September 2020). All women who agrees to participate received clear information on the aims and procedures of the study. All gave their written informed consents.

All patients with live singleton or multiple pregnancies, requiring labor induction for childbirth, with an unfavorable cervical examination (Bishop score < 7), and who were able to complete a questionnaire without help were eligible for inclusion in the study.

To assess the test–retest reliability of the EXIT, all participants were contacted by phone one week after their return home to inform them that they will receive an internet link on this day to complete the questionnaire a second time online within one to two weeks. Respondents who completed the second evaluation (retest) more than three weeks after the first evaluation (test) were excluded from this reliability analysis.

The sample size of the study was determined according to the quality criteria established by COSMIN [25,26]. A sample size of more than 50 subjects is rated good by the COSMIN group for the internal consistency evaluation. A minimum number of 100 subjects or six times the number of items is recommended to ensure satisfactory factor analysis. A sample size of at least 50 subjects is recommended to guarantee an acceptable assessment for reliability.

### 2.4. Data Collection

If they agreed to participate, women self-completed the EXIT between two and four days after delivery.

Sociodemographic and clinical data were recorded from the medical files of patients: maternal age, parity, scarred uterus, multiple pregnancies, gestational age at delivery, indication for IOL, method of IOL, duration of maturative phase, artificial rupture of membranes, epidural analgesia, Bishop score, fetal position at full dilatation, duration to reach 3 cm of dilatation, duration to full dilatation, duration to birth, delivery within 24 h, delivery mode, indication for cesarean section, episiotomy, perineal tear, postpartum hemorrhage (defined as blood loss > 500 mL after delivery), fever during labor (>38.2 °C), manual removal of retained placenta, and maternal–fetal infection. Neonatal data included birth weight, Apgar score < 7 at 5 min, umbilical artery pH, umbilical artery lactates, and neonatal transfer.

An e-mail address was also collected from all women to send them the internet link to complete the questionnaire a second time online for the test–retest.

Study data were collected and managed using REDCap electronic data capture tools hosted at the University Hospital of Clermont-Ferrand [27,28]. REDCap (Research Electronic Data Capture) is a secure, web-based software platform designed to support data capture for research studies.

### 2.5. Statistical Analysis

Statistical analyses were performed with SAS software (version 9.4, SAS Institute, Cary, NC, USA, 2002–2012) and conducted at a two-sided alpha = 0.05 significance level.

Sociodemographic and clinical characteristics of participants were described.

The psychometric properties of the EXIT-French were evaluated for two groups: women who underwent artificial rupture of membranes (ARM group) and women with spontaneous rupture of membranes (SRM group). They consisted of data completeness, factor analysis, internal consistency, descriptive statistics and score distributions, inter-subscale correlations, and convergent validity and reliability.

#### 2.5.1. Data Completeness

The respondent’s acceptability was assessed by looking at the frequency of missing values. Data quality was considered satisfactory if less than 15% of the item data were missing.

#### 2.5.2. Factor Analysis

Factor analysis with an oblique Promax rotation allowing the factors to correlate was performed to study the multidimensionality and distribution of the ten items measuring women’s experiences of IOL [29]. The Kaiser–Meyer–Olkin (KMO) statistic and Bartlett’s test of sphericity were used to check the appropriateness of running the factor analysis. KMO values higher than 0.50 were acceptable [30]. Bartlett’s test requires the yield of a significant result (*p* < 0.05). Eigenvalues higher than 1 (Kaiser criterion) and Cattell’s scree plot [31] were used for factor retention. The solution giving the most adequate factor structure (item loadings greater than 0.32, no or few item cross-loadings, i.e., no or few items with loadings of 0.32 or higher on two or more factors) was retained [29].

Two sets of factor analyses were performed: one without item 6 related to the rupture of membranes in the SRM group and one with this item in the ARM group. 

#### 2.5.3. Internal Consistency

Cronbach’s α coefficient and the Spearman correlation coefficient were used to evaluate the internal consistency of multi-item and two-item subscales, respectively [32]. The minimum required for the coefficient was 0.70 according to the standard used for group comparisons [33].

#### 2.5.4. Descriptive Statistics and Score Distributions

The EXIT-French items and subscale scores’ distributions were described by mean, standard deviation, median, and range. The variability of the EXIT-French scores was investigated for each subscale with the floor and ceiling effects. These effects were considered to be present if more than 15% of the subjects obtained the lowest or highest possible score [34].

#### 2.5.5. Inter-Subscale Correlations

Spearman correlation coefficients were used to evaluate inter-subscale correlations. Correlations were considered very small for coefficients lower than 0.30, small for coefficients between 0.30 and 0.50, moderate from 0.50 to 0.70, and strong if higher than 0.70 [35].

#### 2.5.6. Convergent Validity

Spearman’s coefficients were used to evaluate the correlations between the subscale scores and (1) global satisfaction with the birth experience, (2) likelihood of choosing the same method of IOL again, and (3) likelihood of recommending the method of IOL to a friend or relative. Positive correlations were expected. A negative correlation was expected between the ‘Time taken to give birth’ subscale and the duration between IOL and delivery. Women who gave birth within 24 h and who were multiparous [36] tended to respond more positively (higher scores) in the ‘‘Time taken to give birth’ subscale. The method of induction was expected to be associated with the ‘Experience of subsequent contractions’ subscale [37,38].

#### 2.5.7. Reliability

Stability over time was assessed by the test–retest method. Intraclass correlation coefficients (ICC, absolute agreement, two-way mixed-effect model with single measurement) for subscales were used to estimate reliability. ICC values between 0 and 0.50 indicated poor agreement, between 0.50 and 0.75 moderate agreement, and between 0.75 and 1 good agreement [39].

## 3. Results

### 3.1. Participants

One hundred and sixty-three women were included. The characteristics and clinical outcomes of the participants are described in Table 1. They were nulliparous at 49.7% with a mean age of 30.8 ± 5.5 years (range 16–43) and a mean gestational age of 39.3 ± 1.5 weeks (range 34.1–42). An IOL was indicated for maternal pathologies (28.2%), suspected fetal macrosomia (27.0%), prelabor rupture of membranes (20.9%), prolonged pregnancy (19.0%), diabetes (11.7%), twin pregnancy (4.9%), intrauterine growth restriction (4.3%), fetal pathologies (3.7%), and hypertension and pre-eclampsia (0.6%). More than half of the patients (54.6%) had an artificial rupture of membranes. The majority had an epidural analgesia (95.1%). The delivery occurred within 24 h for 65.4% of patients and a cesarean section was performed for 17.2%.

### 3.2. Data Completeness

The ten items of the EXIT measuring women’s experiences of IOL were completed by 94.4% and 97.3% of the women in the SRM and ARM groups, respectively. The percentage of missing values per item varied between 0 and 1.4% in the SRM group and between 0 and 5.6% in the ARM group. There were no missing values for the other seven optional items.

### 3.3. Factor Analysis

Factor analysis with an orthogonal Promax rotation was performed to study the structure of the items measuring women’s experiences of IOL, first without item 6 related to the rupture of membranes in the SRM group, and then with item 6 in the ARM group. The significance value of Bartlett’s test of sphericity was <0.001 in the two groups (χ2 = 208.0, df = 36 in the SRM group and χ2 = 262.8, df = 45 in the ARM group). The KMO measures of sampling adequacy were 0.583 and 0.606 in SRM and ARM groups, respectively, indicating that the data were suitable for factor analysis. The two-factor analysis identified four factors with eigenvalues greater than one and accounting for 74.0% of the total variance in the SRM group and 73.2% in the ARM group. However, one factor contained only one item (item 5—could move around as freely as wanted to after being induced) and then a three-factor solution was explored in the two groups. In the SRM group, item 5 had loadings greater than 0.32 on the three factors, and in the ARM group, this item had no loading greater than 0.32 on a factor. Then, item 5 was removed and the factor analysis was repeated in the two groups. Two three-factor solutions with eigenvalues greater than one and accounting for 68.7% and 70.0% of the total variance in SRM and ARM groups, respectively, were identified. All items loaded higher than 0.32 on their subscales (Table 2). In the two groups, item 4 loaded higher than 0.32 on two factors and in the SRM group, item 7 loaded higher than 0.32 on two factors. After an evaluation of internal consistency, items 4 and 7 were conserved in factor 3 in the SRM group and item 4 in factor 1 in the ARM group. One of the three subscales identified slightly differed from the original EXIT and two were identical. The first factor in the SRM group and the second one in the ARM group were labeled ‘Experience of subsequent contractions’ and comprised items 8 and 9 as in the original subscale. The second factor in the SRM group and the third one in the ARM group were labeled ‘Time taken to give birth’ and comprised items 1 and 2 as in the original subscale. The third factor in the SRM group and the first one in the ARM group were named ‘Discomfort with IOL’. There were four items in common with the original subscale (items 3, 4, 6, and 7) and one item (item 10) was added to this factor (item removed during analysis of the original EXIT’s structure).

### 3.4. Internal Consistency

In the SRM group, the ‘Time taken to give birth’ and ‘Discomfort with IOL’ subscales did not obtain the minimum required coefficient of 0.70. The Spearman correlation coefficient and Cronbach’s α were, respectively, equal to 0.61 and 0.54. The ‘Experience of subsequent contractions’ subscale showed good internal consistency, with the Spearman correlation coefficient equal to 0.88.

In the ARM group, the ‘Time taken to give birth’ and ‘Experience of subsequent contractions’ subscales showed good internal consistency, with the Spearman correlation coefficients equal to 0.80 and 0.77, respectively. The ‘Discomfort with IOL’ subscale did not obtain the minimum required coefficient of 0.70, having nevertheless a very close Cronbach’s α of 0.67.

### 3.5. Descriptive Statistics, Score Distribution, and Floor and Ceiling Effects

The descriptive statistics and score distributions of the EXIT-French items and subscales are presented by groups in Table 3. ‘Time taken to give birth’ was characterized by a ceiling effect with 20.3% and 15.7% of women in the SRM and ARM groups, respectively, with the highest score. A floor effect was found for the ‘Experience of subsequent contractions’ subscale only in the SRM group with 27.0% of women with the lowest score. Neither floor nor ceiling effects were found for the ‘Discomfort with IOL’ subscale.

### 3.6. Inter-Subscale Correlations

In the SRM group, correlations between the EXIT-French subscales ranged from 0.04 to 0.38. The ‘Time taken to give birth’ subscale had a very small significant correlation with the ‘Discomfort with IOL’ subscale (r = 0.23, *p* = 0.047) and a very small non-significant correlation with the ‘Experience of subsequent contractions’ subscale (r = −0.04, *p* = 0.707). The correlation between the ‘Discomfort with IOL’ and ‘Experience of subsequent contractions’ subscales was small and significant with a coefficient of r = 0.38 (*p* < 0.001).

In the ARM group, correlations between the EXIT-French subscales were very small and non-significant. The Spearman correlation coefficients were r = 0.11 (*p* = 0.293) between the ‘Time taken to give birth’ and ‘Discomfort with IOL’ subscales, r = 0.01 (*p* = 0.948) between the ‘Time taken to give birth’ and ‘Experience of subsequent contractions’ subscales, and r = 0.19 (*p* = 0.074) between the ‘Discomfort with IOL’ and ‘Experience of subsequent contractions’ subscales.

### 3.7. Convergent Validity

As expected, positive correlations were found in the two groups between the EXIT-French subscales and global satisfaction with the birth experience, the likelihood of choosing the same method of IOL again, and the likelihood of recommending the method of IOL to a friend or relative (Table 4). All correlations were significant, except for correlations of global satisfaction with the ‘Experience of subsequent contractions’ subscale in the two groups and with the ‘Discomfort with IOL’ subscale in the ARM group.

The duration between IOL and delivery was significantly correlated to the ‘Time taken to give birth’ subscale with a moderate correlation (r = −0.41, *p* < 0.001 in the SRM group and r = −0.50, *p* < 0.001 in the ARM group). The women who gave birth within 24 h responded more positively in the ‘Time taken to give birth’ subscale (3.9 ± 1.0 versus 2.8 ± 1.3, *p* = 0.002 in the SRM group and 3.7 ± 1.2 versus 2.8 ± 1.1, *p* < 0.001 in the ARM group). Multiparous patients were more positive in the ‘Time taken to give birth’ subscale in the ARM group (3.7 ± 1.1 versus 2.8 ± 1.2, *p* < 0.001).

### 3.8. Reliability

Of the 84 women who completed the second evaluation, 3 returned the retest questionnaire more than three weeks after the first evaluation. For test–retest reliability analysis, 81 women were retained. They were nulliparous at 44.4% with a mean age of 30.7 ± 4.7 years and a mean gestational age of 39.4 ± 1.5 weeks. There were 43 (53.1%) who had an artificial rupture of membranes.

Test–retest reliability was moderate for the ‘Time taken to give birth’ subscale with ICC values of 0.72 (95% CI 0.53–0.85) and 0.61 (95% CI 0.38–0.77) in the SRM and ARM groups, respectively. Reliability was moderate for the ‘Discomfort with IOL’ subscale in the SRM group with an ICC value of 0.59 (95% CI 0.32–0.77) and good in the ARM group with an ICC value of 0.82 (95% CI: 0.69–0.90). In the two groups, the ‘Experience of subsequent contractions’ subscale showed good reliability, with ICC values of 0.87 (95% CI 0.76–0.93) and 0.79 (95% CI 0.64–0.88) in the SRM and ARM groups, respectively.

## 4. Discussion

The present study described the cross-cultural adaptation and evaluation of the psychometric properties of the EXIT-French, including the assessment of the test–retest reliability, which was not explored in the original study.

The EXIT was successfully translated from English to French and adapted to any IOL method. The EXIT-French had good acceptability with very low percentages of missing values per item. It also had good response distribution, indicating that the instrument had been adapted to the studied population. Small floor and ceiling effects were found for, respectively, the ‘Experience of subsequent contractions’ and ‘Time taken to give birth’ subscales.

The three-factor structure of the EXIT-French, found similarly in the SRM and ARM groups, differed slightly from the structure of the original version of the EXIT [22]. As in the original version, item 5 (*could move around as freely as wanted to after being induced*) was removed during the exploratory factor analysis. The ‘Time taken to give birth’ and ‘Experience of subsequent contractions’ subscales were identical to the original subscales. Only the ‘Discomfort with IOL’ subscale differed from the original corresponding subscale. It involved a combination of three or four items from the original subscale and one item that was removed during the factor analysis of the original version of the EXIT. In the French context, unhappiness with the procedures that followed being induced was related to discomfort with IOL. For women who had an artificial rupture of membranes (ARM group), this subscale could contain item 6 as in the original ‘Discomfort with IOL’ subscale. Item 4 (being induced painful) overlapped between the factors related to subsequent contractions and discomfort with IOL in the French version. We opted to place it in the ‘Discomfort with IOL’ subscale as in the original version for better internal consistency of the subscales in the resulting structure.

The internal consistency of the EXIT-French subscales was lower than that of the original subscales [22]. The ‘Time taken to give birth’ subscale in the SRM group and the ‘Discomfort with IOL’ subscale in the SRM and ARM groups did not obtain the minimum required coefficient of 0.70 in our study, having nevertheless a very close coefficient of 0.67 for the ‘Discomfort with IOL’ subscale in the ARM group.

The very small correlations between the EXIT-French subscales in the ARM group implied that they measured relatively different constructs. In the SRM group, the correlation between the ‘Discomfort with IOL’ and ‘Experience of subsequent contractions’ subscales’ was significant but small. These correlations were not explored in the original study.

Convergent validity was explored by assessing the correlations between the subscales and global satisfaction with the birth experience, the likelihood of choosing the same method of IOL again, and the likelihood of recommending the method of IOL to a friend or relative. Positive correlations were found in the two groups as in the original study. As in the original study, the women who experienced a shorter IOL-to-birth interval responded more positively in the ‘Time taken to give birth’ subscale.

The test–retest reliability, which is an essential property [40], showed moderate reliability for the ‘Time taken to give birth’ subscale in the two groups and for the ‘Discomfort with IOL’ subscale in the SRM group, good reliability for the ‘Discomfort with IOL’ subscale in the ARM group, and good reliability for the ‘Experience of subsequent contractions’ subscale in the two groups.

Only nine items in the EXIT-French questionnaire contributed to the subscales, as item 5 was removed during the exploratory factor analysis. So this item and the seven independent items related to global satisfaction and process evaluation could be optional. If time is a priority, only the nine items making up the three subscales could be used as a more succinct instrument.

Our study had some limitations. We did not collect information about the number of women who declined to participate, so no response rate was evaluated. Moreover, the characteristics and clinical outcomes of patients who participated could not be compared to those of patients who did not participate. However, this was not a major bias as our purpose was not to assess women’s experiences of IOL but to study the psychometric properties of the French version of the EXIT. Such an analysis could be minimally impacted by selection bias [41]. For the reliability test, a recommended sample size of at least 50 women could not be included for each group [25,26]. Nevertheless, these psychometric properties, which were not evaluated in the original study [22], were explored in our study and moderate to good ICC were obtained. Further studies are needed on larger samples to confirm the psychometric properties of the EXIT-French. It could also be interesting to test the EXIT-French with different methods of IOL.

## 5. Conclusions

This study provides evidence of the good psychometric properties of the EXIT-French when delivered to women requiring IOL whatever the method of IOL used. The EXIT-French is a valid, reliable, and easy instrument to use for the self-assessment of women’s experiences of IOL in the three weeks following delivery. This tool could be used in research studies as a patient-reported outcome measure to assess the time taken to give birth, discomfort with IOL, and experience of subsequent contractions in three synthetic scores as well as assess global satisfaction and the IOL process in seven optional items. It would also allow the comparison of experiential outcomes across IOL studies in order to include women’s preferences in decisions regarding their care.

## Figures and Tables

**Table 1:** Clinical characteristics of the patients undergoing induction of labor (IOL) and neonatal outcomes.

Characteristics	Variable	*n* = 163
Obstetrical characteristics	Maternal age (years) *	30.8 ± 5.5
	Parity, *n (%)*	
	Nulliparous	81 (49.7)
	Multiparous	82 (50.3)
	Scarred uterus, *n (%)*	8 (4.9)
	Multiple pregnancy, *n (%)*	10 (6.1)
IOL	Gestational age at IOL (weeks) *	39.3 ± 1.5
	Indication of induction, *n (%)*	
	Suspected fetal macrosomia	44 (27.0)
	Gestational diabetes	35 (21.5)
	Prelabor rupture of membranes	34 (20.9)
	Prolonged pregnancy	31 (19.0)
	Maternal pathologies	26 (16.0)
	Twin pregnancy	9 (5.5)
	Intrauterine growth restriction	7 (4.3)
	Fetal pathologies	6 (3.7)
	Pregnancy-induced hypertension	4 (2.5)
	Pre-eclampsia	2 (1.2)
	Method of induction, *n (%)*	
	Oral misoprostol	51 (31.3)
	Vaginal dinoprostone insert	94 (57.7)
	Intracervical ripening balloon	17 (10.4)
	Bishop score at IOL, *n (%)*	
	0–3	120 (73.6)
	4–6	43 (26.4)
	Time IOL—maturative phase (hours) *	12.9 ± 8.9
	Artificial rupture of membranes, *n (%)*	89 (54.6)
	Epidural analgesia, *n (%)*	154 (95.1)
	Time IOL—3 cm of dilatation (hours) *	15.6 ± 10.8
	Time IOL—full dilatation (hours) *	19.3 ± 12.6
	Time IOL—delivery (hours) *	22.1 ± 12.8
Delivery outcomes	Fetal position at full dilatation, *n (%)*	
	Cephalic	92 (97.9)
	Breech	2 (2.1)
	Delivery within 24 h, *n (%)*	106 (65.4)
	Delivery mode, *n (%)*	
	Spontaneous vaginal	119 (73.0)
	Operative vaginal	16 (9.8)
	Cesarean section	28 (17.2)
	Indication for cesarean section, *n (%)*	
	Failure of induction	6 (22.2)
	Failure of dilatation progress	6 (22.2)
	Non-descent of fetal head at full dilatation	4 (14.8)
	Non-reassuring fetal heart rate	8 (29.6)
	Other	3 (11.1)
Maternal issues	Episiotomy, *n (%)*	9 (5.5)
	Perineal tear, *n (%)*	104 (63.8)
	First degree	68 (65.4)
	Second degree	35 (33.7)
	Third and fourth degree	1 (1.0)
	Postpartum hemorrhage > 500mL, *n (%)*	25 (15.3)
	Fever during labor, *n (%)*	7 (4.3)
	Manual removal of retained placenta, *n (%)*	26 (16.0)
	Materno-fetal infection, *n (%)*	5 (3.1)
Neonatal issues	Neonatal weight (g) *	3307.0 ± 581.6
	5-min Apgar score < 7, *n (%)*	6 (3.5)
	Umbilical artery pH *	7.2 ± 0.3
	Umbilical artery lactates *	5.1 ± 2.1
	Neonatal transfer, *n (%)*	12 (7.4)
	Neonatal intensive care unit	9 (5.5)
	Neonatology	3 (1.8)

* Mean ± standard deviation.

**Table 2:** Factor loadings from the factor analysis of EXIT-French items measuring women’s experiences of IOL.

		SRM Group			ARM Group	
	Factor 1	Factor 2	Factor 3	Factor 1	Factor 2	Factor 3
Variance explained (%)	31.6	23.2	13.9	25.1	23.5	21.4
‘Time taken to give birth’ subscale						
1. Happy with how long it took for labor to start after being induced.	0.08	**0.94**	−0.12	0.08	0.00	**0.94**
2. Happy with how long it took for baby to be born after being induced.	−0.06	**0.82**	0.25	−0.02	0.04	**0.95**
‘Discomfort with IOL’ subscale						
3. Unhappy about the number of internal vaginal examinations.	−0.11	0.03	**0.62**	**0.61**	−0.18	0.30
4. Being induced painful.	**0.59**	−0.10	**0.33**	**0.45**	**0.64**	−0.18
6. Having waters broken (membranes ruptured) unpleasant.	-	-	-	**0.77**	0.08	−0.10
7. Experience of unpleasant side effects after being induced.	**0.42**	−0.11	**0.56**	**0.68**	0.23	0.14
10. Unhappy with the procedures that followed being induced.	0.01	0.10	**0.78**	**0.72**	−0.24	−0.08
‘Experience of subsequent contractions’ subscale						
8. Frequency of contractions manageable.	**0.94**	0.07	−0.02	−0.02	**0.89**	0.03
9. Intensity of contractions manageable.	**0.93**	0.04	−0.12	−0.19	**0.90**	0.09

SRM: Spontaneous Rupture of Membranes; ARM: Artificial Rupture of Membranes. Loadings equal to or higher than 0.32 are presented in bold.

**Table 3:** Descriptive statistics and score distributions of the EXIT-French items and subscales.

		Missing Values (%)	Mean ± SD	Range	Median	Floor Effect (%)	Ceiling Effect (%)
SRM group	Item 1	0	3.6 ± 1.2	1.0–5.0	4.0	-	-
	Item 2	1.4	3.6 ± 1.3	1.0–5.0	4.0	-	-
	Item 3 *	0	3.7 ± 1.4	1.0–5.0	4.0	-	-
	Item 4 *	1.4	2.6 ± 1.6	1.0–5.0	2.0	-	-
	Item 5	0	3.9 ± 1.3	1.0–5.0	4.0	-	-
	Item 7 *	0	4.0 ± 1.3	1.0–5.0	4.0	-	-
	Item 8	0	2.4 ± 1.3	1.0–5.0	2.0	-	-
	Item 9	0	2.4 ± 1.3	1.0–5.0	2.0	-	-
	Item 10 *	0	4.3 ± 1.0	1.0–5.0	5.0	-	-
	Time taken to give birth	0	3.6 ± 1.1	1.0–5.0	4.0	5.4	20.3
	Discomfort with IOL	0	3.7 ± 0.8	1.5–5.0	3.8	0	9.5
	Experience of subsequent contractions	0	2.4 ± 1.3	1.0–5.0	2.0	27.0	6.8
ARM group	Item 1	0	3.3 ± 1.3	1.0–5.0	4.0	-	-
	Item 2	1.1	3.4 ± 1.3	1.0–5.0	4.0	-	-
	Item 3 *	1.1	3.8 ± 1.3	1.0–5.0	4.0	-	-
	Item 4 *	0	2.7 ± 1.4	1.0–5.0	2.0	-	-
	Item 5	1.1	3.3 ± 1.3	1.0–5.0	4.0	-	-
	Item 6 *	5.6	4.3 ± 1.1	1.0–5.0	5.0	-	-
	Item 7 *	1.1	3.8 ± 1.3	1.0–5.0	4.0	-	-
	Item 8	0	3.2 ± 1.3	1.0–5.0	4.0	-	-
	Item 9	0	3.0 ± 1.3	1.0–5.0	3.0	-	-
	Item 10 *	0	4.3 ± 1.0	1.0–5.0	5.0	-	-
	Time taken to give birth	0	3.3 ± 1.2	1.0–5.0	3.5	10.1	15.7
	Discomfort with IOL	0	3.8 ± 0.8	1.4–5.0	3.8	0	4.5
	Experience of subsequent contractions	0	3.1 ± 1.2	1.0–5.0	3.5	9.0	7.9

SD: standard deviation; SRM: Spontaneous Rupture of Membranes; ARM: Artificial Rupture of Membranes. * Items with reverse scores.

**Table 4:** Correlations between the EXIT-French subscales and global satisfaction, likelihood of choosing the same method of IOL again, and recommending the method.

		Time Taken to Give Birth	Discomfort with IOL	Experience of Subsequent Contractions
SRM group	Global satisfaction with the birth experience	0.67 ***	0.41 ***	0.14
	Likelihood of choosing the same method of IOL again	0.46 ***	0.50 ***	0.42 ***
	Likelihood of recommending the method of IOL to a friend or relative	0.43 ***	0.42 ***	0.40 ***
ARM group	Global satisfaction with the birth experience	0.46 ***	0.17	0.09
	Likelihood of choosing the same method of IOL again	0.28 **	0.37 ***	0.31 **
	Likelihood of recommending the method of IOL to a friend or relative	0.33 **	0.28 **	0.37 ***

SRM: Spontaneous Rupture of Membranes; ARM: Artificial Rupture of Membranes. Spearman correlation coefficients significantly different from zero: ** *p* < 0.01 and *** *p* < 0.001.

## Data Availability

Not applicable.

## Supplementary Materials

The following supporting information can be downloaded at: https://www.mdpi.com/article/10.3390/jcm11144217/s1, File S1: EXIT-French questionnaire and scoring method.
