# Supplementary material for: Cross-Cultural Adaptation and Psychometric Properties of the French Version of the EXIT to Measure Women’s Experiences of Induction of Labor
Source: J Clin Med. 2022 Jul 20;11(14):4217. doi: 10.3390/jcm11144217 (PMC9317795; doi:10.3390/jcm11144217)
Supplement: Supplementary file 1 [file jcm-11-04217-s001.zip › jcm-1716757-supplementary.pdf]

File S1: EXIT-French questionnaire and scoring method

Evaluation de votre ressenti sur le déclenchement de votre accouchement

Nous vous remercions de prendre quelques minutes pour répondre à ce questionnaire et de nous faire part de votre ressenti sur votre déclenchement et votre accouchement. Nous vous assurons que ce questionnaire est totalement anonyme.

Pour chacune des questions, nous vous remercions de ne cocher qu'une seule case, celle correspondant le mieux à ce que vous avez ressenti.

1. J'ai été satisfaite du temps qu'il a fallu pour que le travail commence après avoir été déclenchée :

☐ Pas du tout d'accord ☐ Pas d'accord ☐ Ni pas d'accord, ni d'accord ☐ D'accord ☐ Tout à fait d'accord

2. J'ai été satisfaite du temps qu'il a fallu pour que mon bébé naisse après avoir été déclenchée :

☐ Pas du tout d'accord ☐ Pas d'accord ☐ Ni pas d'accord, ni d'accord ☐ D'accord ☐ Tout à fait d'accord

3. Je n'ai pas été satisfaite du nombre de touchers vaginaux que j'ai eu :

☐ Pas du tout d'accord ☐ Pas d'accord ☐ Ni pas d'accord, ni d'accord ☐ D'accord ☐ Tout à fait d'accord

4. Etre déclenchée a été douloureux :

☐ Pas du tout d'accord ☐ Pas d'accord ☐ Ni pas d'accord, ni d'accord ☐ D'accord ☐ Tout à fait d'accord

5. J'ai pu me déplacer aussi librement que je le souhaitais après avoir été déclenchée :

☐ Pas du tout d'accord ☐ Pas d'accord ☐ Ni pas d'accord, ni d'accord ☐ D'accord ☐ Tout à fait d'accord

6. La rupture de la poche des eaux par un(e) soignant(e) a été désagréable :

☐ Non concernée (rupture spontanée de la poche des eaux)

☐ Pas du tout d'accord ☐ Pas d'accord ☐ Ni pas d'accord, ni d'accord ☐ D'accord ☐ Tout à fait d'accord

7. J'ai ressenti des effets secondaires désagréables après avoir été déclenchée :

☐ Pas du tout d'accord ☐ Pas d'accord ☐ Ni pas d'accord, ni d'accord ☐ D'accord ☐ Tout à fait d'accord

8. La fréquence de mes contractions était gérable :

☐ Pas du tout d'accord ☐ Pas d'accord ☐ Ni pas d'accord, ni d'accord ☐ D'accord ☐ Tout à fait d'accord

9. L'intensité de mes contractions était gérable :

☐ Pas du tout d'accord ☐ Pas d'accord ☐ Ni pas d'accord, ni d'accord ☐ D'accord ☐ Tout à fait d'accord

10. Je n'ai pas été satisfaite des gestes médicaux qui ont suivi mon déclenchement :

☐ Pas du tout d'accord ☐ Pas d'accord ☐ Ni pas d'accord, ni d'accord ☐ D'accord ☐ Tout à fait d'accord

11. Dans l'ensemble, j'ai été satisfaite de mon accouchement :

☐ Pas du tout d'accord ☐ Pas d'accord ☐ Ni pas d'accord, ni d'accord ☐ D'accord ☐ Tout à fait d'accord

12. Avec le recul, avez-vous le sentiment que vous étiez bien préparée pour le déclenchement ?

☐ Non, absolument pas ☐ Plutôt non ☐ Ni oui, ni non ☐ Plutôt oui ☐ Oui, tout à fait

13. Avec le recul, avez-vous le sentiment que tout ce qui s'est passé pendant votre accouchement était nécessaire ?

☐ Non, absolument pas ☐ Plutôt non ☐ Ni oui, ni non ☐ Plutôt oui ☐ Oui, tout à fait

14. Compte tenu de votre expérience, choisiriez-vous d'être à nouveau déclenchée de cette façon ?

☐ Non, absolument pas ☐ Plutôt non ☐ Ni oui, ni non ☐ Plutôt oui ☐ Oui, tout à fait

15. Recommanderiez-vous à une amie ou à un proche d'être déclenchée de cette façon ?

☐ Non, absolument pas ☐ Plutôt non ☐ Ni oui, ni non ☐ Plutôt oui ☐ Oui, tout à fait

16. Pour finir, y a-t-il autre chose que vous aimeriez partager sur votre vécu du déclenchement et de l'accouchement ?

……………………………………………………………………………………………………......

……………………………………………………………………………………………………......

……………………………………………………………………………………………………......

……………………………………………………………………………………………………......

……………………………………………………………………………………………………......

……………………………………………………………………………………………………......

17. Avez-vous des suggestions pour améliorer la façon dont les femmes vivent leur déclenchement ?

……………………………………………………………………………………………………......

……………………………………………………………………………………………………......

……………………………………………………………………………………………………......

……………………………………………………………………………………………………......

……………………………………………………………………………………………………......

……………………………………………………………………………………………………......

Nous vous remercions pour votre participation.

The EXIT-French is a self-administered questionnaire containing 10 items assessing women's experience of induction of labor (items 1 to 10) and 7 optional independent items related to global satisfaction and process evaluation (items 11 to 17). Item 6 is addressed only to women who underwent artificial rupture of membranes (ARM group).

Items 1 to 10 assessing women's experience of induction of labor (IOL) are scored on a 5-point Likert scale ranging from 1 (strongly disagree) to 5 (strongly agree). Five of the ten items have reverse-scores (items 3, 4, 6, 7 and 10) so that a score of 1 represents a negative experience and a score of 5 represents a positive experience.

Three subscales comprised 9 of these 10 items: 'Time taken to give birth', 'Discomfort with IOL' and 'Experience of subsequent contractions'. Each subscale is scored separately by calculating the mean of the individual scores of the items listed in the subscale. Scores of the subscales range from 1 (negative experience) to 5 (positive experience)

Scoring method:

- Time taken to give birth: mean of the scores of items 1 & 2.
- Experience of subsequent contractions': mean of the scores of items 8 & 9.
- Discomfort with IOL: mean of the scores of items 3, 4, 7 & 10 (*).
- Discomfort with IOL – ARM group: mean of the scores of items 3, 4, 6, 7 & 10 (*).

(*) *Reverse-scores: 1 (strongly agree) to 5 (strongly disagree)*
